# Does Q.Clear Processing Change PET Ratios? Quantitative Evidence Using BTXBrain-DAT

**DOI:** 10.3390/brainsci15101036

**Published:** 2025-09-24

**Authors:** Ari Chong, Jung-Min Ha, Ji Yeon Chung

**Affiliations:** 1 Department of Nuclear Medicine, College of Medicine, Chosun University, Gwangju 61452, Republic of Korea; arichong.md@gmail.com; 2 Department of Neurology, College of Medicine, Chosun University, Gwangju 61452, Republic of Korea; jiyeoni@chosun.ac.kr

**Keywords:** dopamine transporter, positron-emission tomography (PET), image enhancement, quantitative analysis, Parkinson’s disease

## Abstract

**Introduction**: Bayesian penalized likelihood (BPL) reconstruction algorithms, commercially implemented as Q.Clear (GE Healthcare), enhance image quality but may alter quantitative metrics. The impact of BPL on dopamine transporter (DAT) PET quantification, including ratios, remains unclear. This study investigates whether Q.Clear processing alters key metrics such as specific binding ratios (SBRs) and interregional ratios. **Methods**: We retrospectively analyzed 170 paired F-18 FP-CIT PET datasets reconstructed with conventional 3D-OSEM (baseline-DICOM) and Q.Clear (Q.Clear-DICOM). Quantification was performed using BTXBrain-DAT (Brightonix Imaging), yielding 57 specific binding ratios (SBRs), three asymmetry indices, and nine interregional ratios. Paired statistical tests, Bland–Altman plots, and reproducibility checks were conducted. Visual reads by two nuclear medicine physicians were also compared between datasets. **Results**: Q.Clear processing significantly altered all quantitative metrics (*p* < 0.001). SBR values changed in all 57 regions, with most high-uptake regions showing an increase and low-uptake regions showing a decrease. Striatal and caudate asymmetry indices showed significant differences (*p* < 0.0001), whereas the putamen index remained stable. All interregional ratios differed significantly, although Bland–Altman analysis indicated relative stability for ratios compared with asymmetric indices. BTXBrain-DAT showed perfect reproducibility on repeat analysis, and visual interpretation was unaffected by reconstruction method. **Conclusions**: Q.Clear (BPL) reconstruction substantially influences F-18 FP-CIT PET quantification, including ratios and asymmetry indices, while leaving visual interpretation unchanged. These findings highlight the need for caution when using image enhancement functions for quantitative analysis, particularly in clinical studies involving low-uptake regions or multicenter data comparisons.

## 1. Introduction

The dopamine transporter (DAT) is located at the terminals of dopaminergic neurons, where it plays a key role in reabsorbing dopamine released into the synapse. The primary pathogenesis of Parkinson’s disease (PD) involves the loss of dopaminergic neurons located in the substantia nigra of the midbrain. DAT imaging is widely recognized as a biomarker that effectively mirrors this process [1]. Presynaptic striatal DAT binding shows a strong correlation with the availability of dopaminergic neurons [2,3]. DAT imaging, including fluorine-18 labeled N-3-fluoropropyl-2β-carbomethoxy-3β-(4-iodophenyl) nortropane (F-18 FP-CIT) positron emission tomography (PET), has proven to be highly sensitive in detecting dopaminergic system degeneration and is approved for clinical use [2,3,4,5].

The F-18 FP-CIT PET is primarily utilized for visual interpretation. In normal subjects, the typical imaging findings display a rabbit-like uptake pattern on maximum-intensity-projection (MIP) images, characterized by symmetric and homogeneous DAT binding in both striata [6]. However, if abnormalities are identified, it is crucial to localize the lesions, evaluate asymmetry, and determine whether the reduced DAT binding is diffuse or focal [6]. Additionally, quantitative analysis is employed, with various methods, such as inter-subregional ratios or asymmetric indices, being actively studied [7,8,9,10,11].

Recent advancements in imaging technology have prompted various manufacturers to develop programs aimed at enhancing image quality. As part of these efforts, Bayesian penalized likelihood (BPL) iterative reconstruction, commercially implemented as Q.Clear (GE Healthcare), has been the latest PET/CT scanner. This algorithm adjusts background noise textures in images based on activity levels [12]. This algorithm enhances image quality by producing clearer and sharper images. However, numerous studies have reported changes in quantitative metrics, such as SUV values, after the application of BPL reconstruction [13,14,15,16]. Consequently, its use in quantitative research, especially in multicenter studies, is discouraged due to the need for caution.

In images enhanced with quality improvement functions, it is generally expected that variations in quantitative analysis values will occur. However, for metrics such as left-right asymmetry indices or interregional uptake ratios, these ‘ratios’ might remain relatively stable because they are inherently comparative measures. This raises an important question: could interregional ratios, by their nature, be largely unaffected by these enhancements? This consideration formed the starting point for this study, yet no prior research has systematically investigated this phenomenon.

Unlike oncologic PET, where the effects of BPL (Q.Clear) reconstruction have been extensively studied, its impact on brain PET quantification—particularly in dopamine transporter (DAT) imaging—remains largely unexplored. Therefore, this study aims to compare and analyze whether commonly used quantitative metrics, such as asymmetric indices and interregional ratios, differ between PET images processed with the GE Q.Clear (Q.Clear-DICOM) image enhancement algorithm and those reconstructed with conventional three dimensional ordered-subset expectation maximization (3D-OSEM; baseline-DICOM), using the quantification software BTXBrain version 1.1.1 (Brightonix Imaging Inc., Seoul, Republic of Korea), to better understand the effects of image enhancement on quantitative analyses.

## 2. Materials and Methods

### 2.1. Study Design

This retrospective study analyzed F-18 FP-CIT PET/CT images acquired at our institution between 28 September 2020 and May 2024. This retrospective dataset included 170 consecutive F-18 FP-CIT PET scans performed for suspected Parkinsonism. The cohort was heterogeneous, comprising both normal and abnormal DAT uptake patterns on visual interpretation (78 abnormal, 92 normal). Visual reads were conducted independently of quantitative analyses. A total of 170 paired datasets, each consisting of a Q.Clear-DICOM and a baseline DICOM, were retrieved from the nuclear medicine department’s miniPACS for analysis. The Q.Clear-DICOM images were reconstructed using GE Q.Clear, a post-processing algorithm integrated into the PET/CT system. The baseline-DICOM images were reconstructed with conventional 3D-OSEM. Both datasets were analyzed using BTXBrain-DAT software version 1.1.1 (Brightonix Imaging Inc., Seoul, Republic of Korea) [17]. Quantification variables obtained from the Q.Clear-DICOM and baseline-DICOM datasets were compared using paired statistical tests to evaluate the effects of Q.Clear processing. To evaluate the reproducibility of the BTXBrain-DAT software, the baseline-DICOM dataset was analyzed twice, approximately one month apart, by the same user. The software settings were identical for both analyses. In addition to quantitative analysis, all reconstructed F-18 FP-CIT PET images (Q.Clear and conventional 3D-OSEM) were visually reviewed by two board-certified nuclear medicine physicians to determine whether reconstruction affected clinical interpretation.

All data used in this study were fully anonymized, containing no patient-identifiable information. DICOM files were processed in a de-identified manner to ensure compliance with data privacy regulations.

### 2.2. Imaging Protocol: PET/CT Scanning

F-18 FP-CIT PET/CT scans were performed using a Discovery MI DR PET/CT scanner (GE Healthcare Waukesha, WI, USA). Patients were injected intravenously with 5 mCi (185 MBq) of F-18 FP-CIT, and imaging was initiated 90 min post-injection. Each scan lasted for 15 min, with patients positioned supine and head-first on the scanning table.

The PET images were acquired with a matrix size of 256 × 256 and a field of view (FOV) of 25 cm, while CT images were obtained with a matrix size of 512 × 512 and an FOV of 25 cm. The CT parameters included a tube voltage of 120 kV, a tube current of 200 mA, a rotation time of 0.5 s, a helical thickness of 2.5 mm, and a pitch of 0.531:1. The detector coverage was 20 mm. PET image reconstruction was performed using two methods: the baseline-DICOM, reconstructed with 3D-OSEM (VPHD) using 4 iterations, 32 subsets, and a 4.0 mm cut-off; and the Q.Clear-DICOM, reconstructed with GE’s Q.Clear algorithm (β = 300), incorporating point spread function (PSF) and time-of-flight (TOF) corrections. The β = 300 setting was selected after initial internal testing of multiple β values and has since been used as the institutional standard, as it provided the most consistent image quality. CT reconstruction utilized standard protocols with MAR (Metal Artifact Reduction), 100/35 kernel, and ASIR-V AR50 settings. PET and CT images were co-registered, and attenuation correction was applied based on the CT data.

### 2.3. Data Analysis Using BTXBrain-DAT

Anonymized DICOM pairs (Q.Clear-DICOM and baseline-DICOM) were quantified using BTXBrain-DAT software [17]. This commercial AI-based software provides automated quantitative values by directly processing reconstructed DICOM images, without the need for anatomical images such as MRI. In this software, volumes-of-interest (VOIs) are automatically segmented based on atlas templates from the Melbourne Subcortical Atlas, with the occipital cortex as the reference region. No manual or semi-automatic adjustments were performed, eliminating inter-observer variability. Quantification outputs were calculated as Specific Binding Ratios (SBRs). The SBR methodology follows established guidelines, including the EANM guidelines [18].

### 2.4. Variables for Comparison

Each quantification analysis using BTXBrain-DAT produced values for 19 subregions, 3 asymmetry indices, and 3 ratios. For subregions, SBRs were obtained separately for total, right, and left regions, resulting in 57 variables (19 subregions × 3 regions). The three asymmetry indices (striatal asymmetry index, caudate asymmetry index, and putamen asymmetry index) were identical across total, right, and left hemispheres, providing 3 variables. For ratios, three types were analyzed: putamen-to-caudate ratio, caudate-to-putamen ratio, and anterior-to-posterior putamen ratio. These were calculated for total, right, and left regions, yielding 9 variables (3 ratios × 3 regions). Thus, a total of 69 variables were generated per dataset, forming paired sets for comparison between Q.Clear-DICOM and baseline-DICOM.

### 2.5. Statistical Analysis

**Figure 1:**
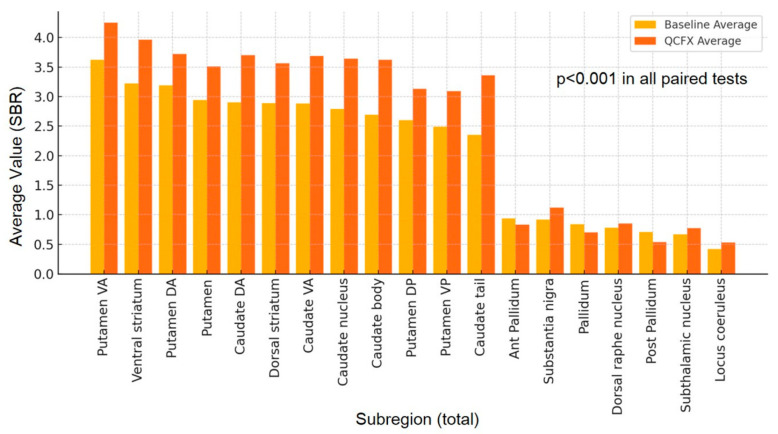
Comparison of Total Lesion Values between Baseline and Q.Clear-DICOM across 19 Subregions. The graph illustrates average values to depict overall trends. Subregions are arranged in descending order based on their average Specific Binding Ratio (SBR) values in the baseline DICOM. All pairs showed statistically significant differences in paired tests (*p* < 0.001).

**Figure 2:**
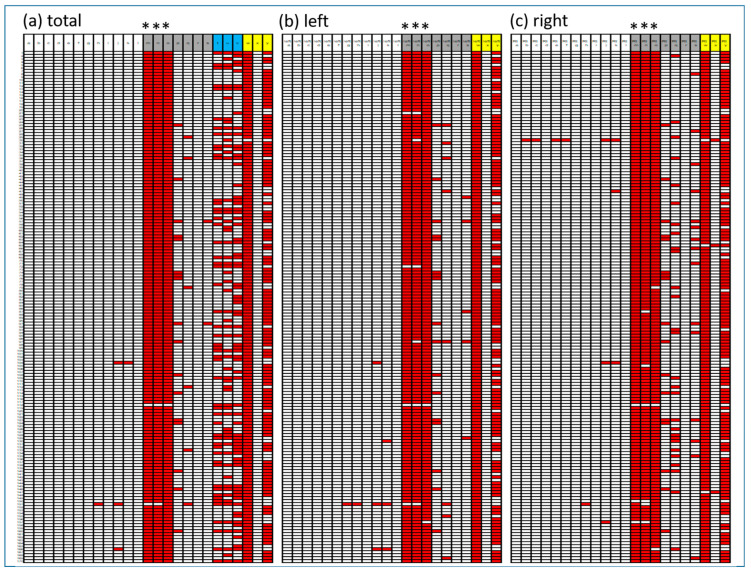
Differences Between Quantification Values: Q.Clear-DICOM Minus Baseline-DICOM (Negative Values Indicated as Red Cells). This figure illustrates the differences between Q.Clear-DICOM and Baseline-DICOM quantification values across 170 analysis pairs. Each row corresponds to a single comparison pair, and the rows are consistent across (**a**–**c**) panels. Columns represent individual variables. The red cells within the matrix indicate negative differences, highlighting areas where quantification values obtained using Q.Clear-DICOM are lower than those from Baseline-DICOM. Rows are ordered randomly, ensuring unbiased visualization. Panel (**a**) displays results for the total dataset, while panels (**b**,**c**) represent left and right regions, respectively. Column headings denoted by letters correspond to specific brain regions or indices as follows: a–l (white heading): Specific Binding Ratio (SBR) values for the following regions: a, Dorsal striatum; b, Caudate nucleus; c, Caudate DA; d, Caudate VA; e, Caudate tail; f, Caudate body; g, Putamen; h, Putamen DA; i, Putamen VA; j, Putamen DP; k, Putamen VP; l, Ventral striatum. m–s (gray heading, areas with an average SBR < 1 based on baseline-DICOM total areas): m, Total_Pallidum; n, Ant Pallidum; o, Post Pallidum; p, Dorsal raphe nucleus; q, Locus coeruleus; r, Substantia nigra; s, Subthalamic nucleus. t–v (blue heading): t, Striatal asymmetry index; u, Caudate asymmetry index; v, Putamen asymmetry index. w–y (yellow heading): w, Total_Putamen-caudate ratio; x, Total_Caudate-putamen ratio; y, Total_Anterior-posterior. Variables marked with an asterisk (***)—Total Pallidum (l), Anterior Pallidum (m), and Posterior Pallidum (n).

To assess overall changes in SBR values between baseline-DICOM and Q.Clear-DICOM, we plotted the average values of subregions (Figure 1) and summarized the distribution of total area values (Table 1). We also calculated paired differences and identified negative values to further characterize the data distribution (Figure 2). Normality was tested using the Kolmogorov–Smirnov (K–S) test [19]. Paired comparisons between baseline variables and Q.Clear-DICOM variables were conducted. For normally distributed variables, paired t-tests were performed (IBM SPSS Statistics version 29.0.1.0). For non-normally distributed variables (Putamen, Putamen DA, Putamen DP, Putamen VP, Posterior Pallidum, Dorsal Raphe Nucleus, and Locus Coeruleus), the Wilcoxon signed-rank test was applied (MedCalc Software Ltd. version 20.007 (Ostend, Belgium)). Agreement between baseline- and Q.Clear-reconstructed values was further evaluated using Bland–Altman plots.

## 3. Results

### 3.1. Differences in 19 Subregion Variables

Paired tests revealed significant differences (*p* < 0.001) in SBRs across all 57 subregion variables between baseline and Q.Clear datasets. Table 1 and Figure 1 present the distribution of total area SBR values across Baseline and Q.Clear-DICOM datasets. Figure 2 illustrates the differences in quantification values, where red cells indicate cases where Q.Clear-DICOM values are lower than baseline-DICOM values. Among the analyzed regions, variables such as dorsal striatum (a), caudate VA (d), putamen (g), putamen VA (i), ventral striatum (o), and substantia nigra (r) consistently exhibited higher values in Q.Clear-DICOM compared to baseline-DICOM, achieving a 100% positive difference rate. While most regions showed higher SBR values with Q.Clear processing, this trend was not universal. Notably, variables marked with an asterisk (***)—Total Pallidum (l), Anterior Pallidum (m), and Posterior Pallidum (n)—demonstrated the highest proportion of negative differences across all regions, as indicated by the dense concentration of red cells in these columns. This pattern may reflect region-specific characteristics or unique algorithmic effects of the Q.Clear processing.

**Figure 3:**
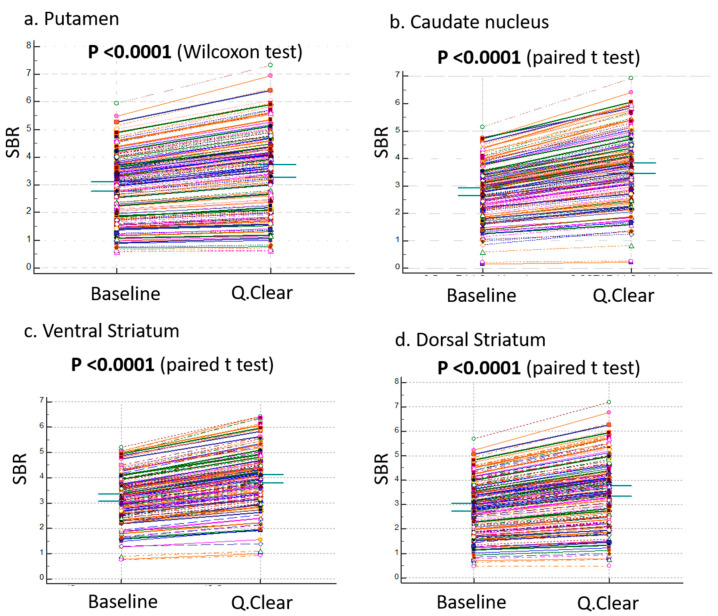
Comparisons of Regional Variables Between Baseline and Q.Clear Variables. This figure illustrates the comparisons of regional variables between the baseline-DICOM and Q.Clear-DICOM datasets, focusing on four representative regions among the 19 subregions analyzed: (**a**) putamen, (**b**) caudate nucleus, (**c**) ventral striatum, and (**d**) dorsal striatum (all representing total regions). Significant differences (<0.001) were observed across all 19 subregions. The vertical axis represents the Specific Binding Ratio (SBR).

In contrast, Figure 3 highlights the comparisons in four representative regions: putamen (a), caudate nucleus (b), ventral striatum (c), and dorsal striatum (d), all representing total regions. In these regions, Q.Clear-DICOM consistently exhibited significantly higher SBR values compared to baseline-DICOM (*p* < 0.001). This suggests that the Q.Clear processing algorithm generally enhances quantification values in these selected areas. However, as shown in the broader dataset from Figure 2, not all regions follow this increasing trend, underscoring the variability in Q.Clear’s effects on different brain regions.

### 3.2. Comparison of Three Asymmetric Indices

**Figure 4:**
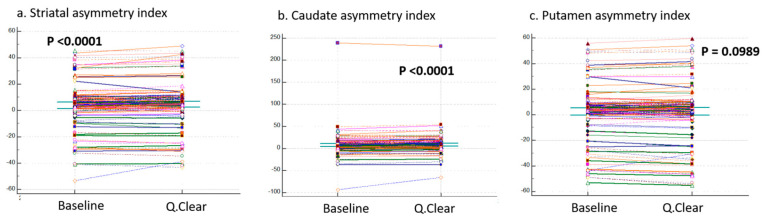
Comparisons of Three Indexes Between Baseline and Q.Clear Variables. This figure compares three indexes—(**a**) striatal asymmetry index, (**b**) caudate asymmetry index, and (**c**) putamen asymmetry index—between the baseline-DICOM and Q.Clear-DICOM datasets. Significant differences were observed for the striatal asymmetry index and caudate asymmetry index between the two groups, while the putamen asymmetry index showed no statistically significant difference. Unlike regional variables, these indexes are calculated globally without distinguishing between **total**, **left**, and **right** regions. Therefore, the values for each index are identical across all panels.

The blue columns in Figure 2 represent the values of the three asymmetric indices: striatal asymmetry index, caudate asymmetry index, and putamen asymmetry index. Since all three indices failed to meet the assumption of normality, the Wilcoxon signed-rank test was used for paired comparisons. Significant differences were observed for the striatal asymmetry index and caudate asymmetry index between baseline and Q.Clear variables (*p* < 0.0001). However, no significant difference was found for the putamen asymmetry index (*p* = 0.0989). These results are summarized in Figure 4. As shown, the asymmetric indices obtained from Q.Clear-DICOM do not consistently exhibit higher or lower values compared to those obtained from baseline-DICOM.

### 3.3. Comparison of Three Ratio Variables

The yellow columns in Figure 2 represent the values of three ratios: the putamen-caudate ratio, caudate-putamen ratio, and anterior–posterior putamen ratio. Panels (a), (b), and (c) correspond to total, left, and right regions, respectively. In the yellow columns, the first row corresponds to the total putamen-caudate ratio. While not 100%, the vast majority of cells are red (left: 100%, total: all but one, right: all but three), indicating that Q.Clear-DICOM values are generally lower than baseline-DICOM values. The second row, representing the caudate-putamen ratio, shows that Q.Clear-DICOM values are predominantly higher. The anterior–posterior putamen ratio demonstrates a more variable pattern, with no consistent trend across regions.

**Figure 5:**
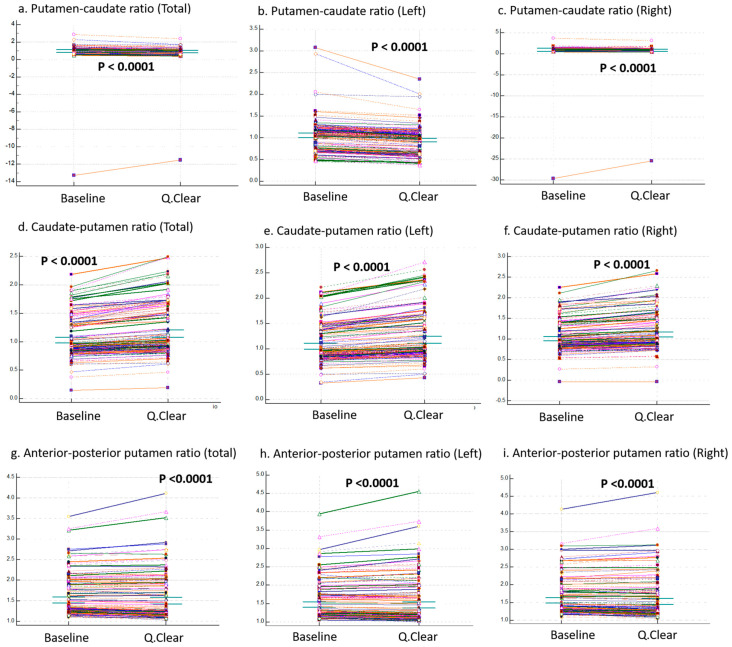
Comparisons of Ratios Between Baseline and Q.Clear Variables. This figure compares three ratios—(**a**–**c**) putamen-to-caudate ratio, (**d**–**f**) caudate-to-putamen ratio, and (**g**–**i**) anterior-to-posterior putamen ratio—between baseline-DICOM and Q.Clear-DICOM datasets. These ratios were calculated separately for **total**, **left**, and **right** regions, and the corresponding values are displayed in each panel. Significant differences were observed between baseline and Q.Clear variables for all ratios across total, left, and right regions. This consistent pattern highlights the impact of Q.Clear processing on these derived ratios, reflecting potential regional variations or adjustments introduced by the algorithm.

Figure 5 presents the results of paired tests, showing significant differences in all three ratios (putamen-caudate ratio, caudate-putamen ratio, and anterior–posterior putamen ratio) across total, left, and right regions between baseline and Q.Clear variables (*p* < 0.0001).

**Figure 6:**
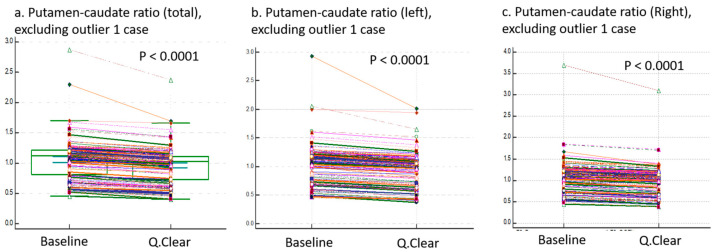
Comparisons of Putamen-to-Caudate Ratios Between Baseline and Q.Clear Variables, Excluding Outlier Case. This figure presents putamen-to-caudate ratios of (**a**) **total**, (**b**) **left**, and (**c**) **right** regions derived from baseline-DICOM and Q.Clear-DICOM datasets after excluding one outlier case. Significant differences (*p* < 0.0001) were observed across all regions, consistent with the patterns described in Figure 5. These results further highlight the impact of Q.Clear processing on regional ratio calculations, even after accounting for outlier effects.

To address potential data distortion caused by an identified outlier, Figure 6 provides a visual representation of the putamen-caudate ratio (total, left, and right) after excluding this outlier. Importantly, the outlier did not affect the statistical significance or general trends of the results.

### 3.4. Bland–Altman Plots

**Figure 7:**
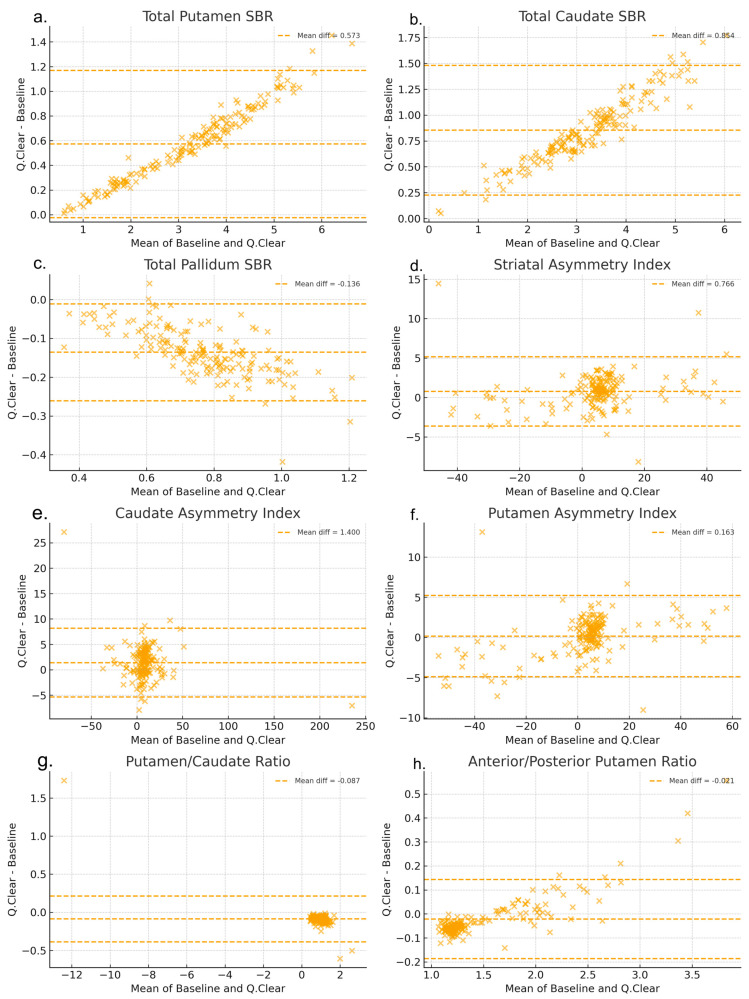
Bland–Altman plots of representative quantitative metrics comparing Q.Clear (BPL reconstruction) and baseline (conventional 3D-OSEM). Panels show: (**a**) Total Putamen SBR, (**b**) Total Caudate SBR, (**c**) Total Pallidum SBR, (**d**) Striatal asymmetry index, (**e**) Caudate asymmetry index, (**f**) Putamen asymmetry index, (**g**) Putamen-to-Caudate ratio (total), and (**h**) Anterior-to-Posterior putamen ratio (total). Solid lines indicate mean bias, and dashed lines represent 95% limits of agreement. Detailed interpretation is provided in the Results section.

Figure 7 compares quantitative metrics between baseline-DICOM and Q.Clear DICOM using Bland–Altman plots. Putamen exhibited proportional bias, with greater differences at higher baseline values, while Pallidum showed a constant negative bias across the range. Ratios (putamen–caudate and anterior–posterior) demonstrated relative stability, whereas asymmetry indices, particularly striatal and putamen asymmetry, displayed wider variability.

### 3.5. Evaluation of BTX Program Reproducibility

To evaluate the reproducibility of the BTXBrain-DAT program, the same baseline-DICOM dataset was analyzed twice, approximately one month apart. The quantification results from the first analysis were referred to as baseline variables 1, and those from the second analysis as baseline variables 2. All variables were identical between baseline variables 1 and baseline variables 2. The differences between the paired variables were zero for all 69 variables, demonstrating perfect consistency between the two analyses.

### 3.6. Effect on Visual Interpretation

**Figure 8:**
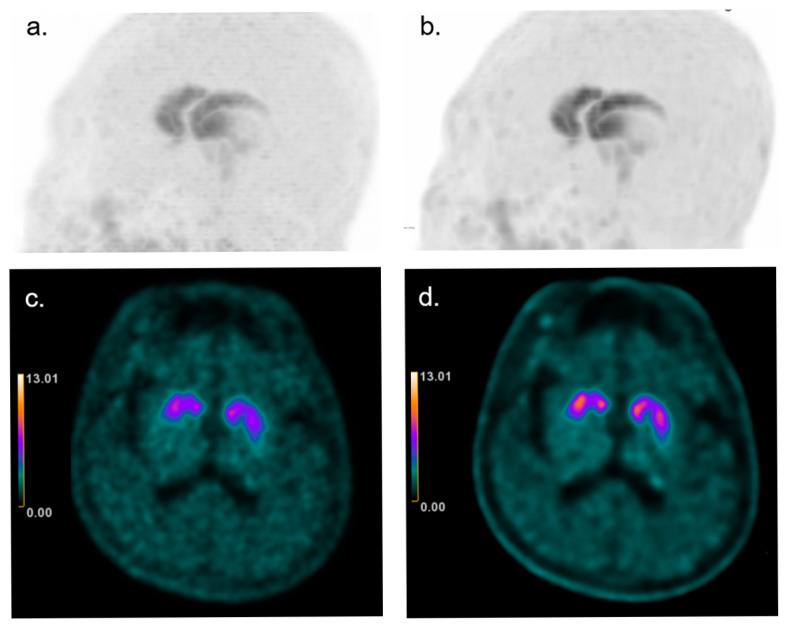
Representative abnormal F-18 FP-CIT PET images reconstructed with baseline (conventional 3D-OSEM; panel (**a**,**c**)) and Q.Clear images (BPL reconstruction; panel (**b**,**d**)). Maximum intensity projections (**a**,**b**) and axial slices in GE color scale (**c**,**d**) are shown. Q.Clear reconstruction demonstrates reduced background noise and sharper striatal uptake contours, while the overall clinical interpretation remained unchanged.

No change in clinical interpretation was observed between Q.Clear-reconstructed and conventional F-18 FP-CIT PET images (Figure 8).

## 4. Discussion

To our knowledge, this is the first study to demonstrate that Bayesian penalized likelihood (BPL) reconstruction, commercially implemented as Q.Clear, alters not only specific binding ratios (SBRs) but also derived indices such as asymmetry and interregional ratios in F-18 FP-CIT PET. Using BTXBrain-DAT, we found that BPL processing affected quantitative values across multiple brain regions as well as ratio-based metrics, indicating its broad influence on both absolute and derived measures. Notably, the significant alteration in asymmetric indices, such as the striatal asymmetry index, was unexpected and highlights the particular vulnerability of these metrics to reconstruction algorithms.

As anticipated, regions with high FP-CIT uptake (SBR > 1) showed increased quantitative values following BPL processing (Figure 1; white headings a–l in Figure 2). However, regions with lower FP-CIT uptake, such as the Pallidum, Substantia Nigra, and Dorsal Raphe Nucleus, demonstrated decreased SBR values after processing (Figure 1; gray headings in Figure 2). This pattern highlights the differential effect of BPL, which tends to enhance high signals while suppressing lower ones.

BPL algorithms effectively reduce noise and enhance signal-to-noise ratio (SNR) [12], but this smoothing process can differentially affect high- and low-uptake regions, potentially exaggerating or minimizing asymmetries. Asymmetry indices are inherently sensitive to small variations in pixel values, which may explain the observed changes [20]. Previous studies have also shown that BPL parameter selection, such as the γ and β values, can influence image quality and quantification accuracy [21,22]. In our study, although BPL reconstruction improved image sharpness and reduced background noise (Figure 8), these changes did not alter lesion detectability or the outcome of clinical visual interpretation.

In contrast to the significant changes observed in striatal and caudate asymmetry indices, the putamen asymmetry index did not differ significantly before and after BPL processing. This stability may reflect the putamen’s relatively strong signal and larger size, which reduce susceptibility to noise-related variability. These findings highlight regional variability in the impact of BPL processing, suggesting that both baseline signal intensity and ROI size influence the sensitivity of asymmetry indices to reconstruction algorithms.

The pallidum, a relatively small region with low DAT density, exhibited consistent decreases in SBR values post-BPL processing (Figure 2; red cells). Its small size and low signal make it particularly vulnerable to noise and algorithmic biases during quantification. While not directly reported, some studies have implied that smoothing effects could influence pixel value distortion at boundaries [23,24,25]. Although absolute differences were generally larger in high-uptake regions, the relative impact of BPL processing appears more critical in low-uptake areas, where even modest absolute deviations can translate into disproportionately large relative changes. Interestingly, this phenomenon was not observed in other small low-uptake regions such as the dorsal raphe nucleus, substantia nigra, or locus coeruleus, possibly because their uniformly low background environments and irregular ROIs reduce the visibility of consistent reconstruction-related biases compared with the larger, well-defined pallidum.

Previous research using I-123 FP-CIT ENC-DAT SPECT imaging also reported significant variability in SBR values depending on reconstruction methods and phantom calibration, cautioning against inter-center data comparisons [26]. However, our study uniquely demonstrates that BPL reconstruction affects not only SBR values but also ratios and asymmetric indices in FP-CIT PET imaging. This underscores the importance of evaluating algorithm-specific effects on all quantitative metrics.

Bland–Altman analyses (Figure 7) clarified the patterns of reconstruction-related bias. Putamen SBR showed proportional bias, with larger deviations at higher baseline values, a phenomenon also reported in oncologic PET [27], suggesting that it is not unique to DAT imaging. In our data, pallidum showed a relatively small but consistent negative bias, indicating that reconstruction effects may vary depending on regional characteristics such as size and uptake level. Asymmetry indices, particularly striatal and putamen asymmetry, displayed wider variability, indicating their potential vulnerability to reconstruction conditions. In contrast, interregional ratios (putamen-to-caudate and anterior-to-posterior) appeared relatively stable in Bland–Altman plots, although paired statistical tests still revealed significant differences between baseline and BPL reconstructions (Figure 7).

These findings align partly with previous reports using non-DAT tracers. Lindström et al. [28] demonstrated in F-18 flutemetamol and F-18 FDG PET that SUVR and z-scores could vary depending on reconstruction methods and the choice of reference region, consistent with the variability we observed. By contrast, Wagatsuma et al. [29] reported that SUVR remained relatively stable across different BPL parameter settings. Taken together, these prior results suggest that tracer characteristics and reference region selection strongly influence the degree to which reconstruction alters quantitative metrics. In our FP-CIT study, the vulnerability of subcortical regions with lower uptake (e.g., pallidum) may partly explain why ratios and indices were not uniformly stable across all metrics. The consistent decrease in pallidal uptake observed in our study may be partly explained by the point-spread-function (PSF) correction embedded in the BPL reconstruction. PSF-based reconstructions are known to influence boundary regions due to edge effects [23]. By sharpening striatal uptake, PSF correction may reduce signals in adjacent peripheral regions such as the globus pallidus. Further investigation using 3D-OSEM with PSF correction would be valuable to confirm whether this effect is algorithm-specific or a general consequence of PSF modeling.

BTXBrain-DAT, an AI-based PET quantification software, was used for ROI definition in this study. Unlike MRI-based segmentation approaches, BTXBrain-DAT delineates ROIs directly from PET signals. Although this method has shown high concordance with gold-standard techniques in prior amyloid studies [30,31], BPL-induced signal changes may alter ROI boundaries, particularly in low-uptake regions such as the pallidum where boundaries are indistinct. Validation with anatomical template-based methods is therefore warranted to confirm these findings.

The observed changes in asymmetric indices and ratios could have diagnostic implications, as these metrics are often used to differentiate neurodegenerative diseases. However, diagnostic thresholds for these indices vary across centers due to differences in equipment, patient physiology, and image processing methods. The clinical significance of these changes remains unclear, and further studies are needed to assess their impact on patient care. Although visual assessment was beyond the primary aim of this study, we reviewed all reconstructed images purely based on image appearance to evaluate potential effects on clinical interpretation. The improved image quality with BPL reconstruction (Q.Clear) did not change the outcome of clinical visual reads.

This study has several limitations. First, all evaluations were conducted using BTXBrain-DAT, which does not utilize MRI-based segmentation. Reanalysis with other quantification tools, such as Statistical Parametric Mapping (SPM), is recommended, particularly for regions like the pallidum. Second, the specific BPL parameters used (e.g., β values) may have influenced the results, as previous study has shown that parameter selection affects quantification [14]. Third, the generalizability of these findings to other radiotracers is uncertain and requires further investigation. Fourth, this was a single-center study with a relatively small sample size, which may limit the generalizability of our findings. Fifth, we did not evaluate direct clinical correlations (e.g., UPDRS scores or final diagnoses), and this should be addressed in future studies. Finally, cost-effectiveness aspects of PET imaging in parkinsonian syndromes, as recently discussed by Fezeu et al. [32], were not assessed in our study.

## 5. Conclusions

To our knowledge, this is the first study to demonstrate that Bayesian penalized likelihood (BPL) reconstruction (Q.Clear) alters not only SBR values but also derived DAT PET metrics such as asymmetry indices and interregional ratios. Although improved image quality with Q.Clear did not affect visual interpretation, our findings provide important insights into the potential challenges of interpreting quantitative PET data. These results underscore the need for methodological consistency and standardization, particularly in multicenter studies. To ensure reliable and reproducible outcomes, baseline- and BPL-processed datasets should not be mixed within a single study.

## Figures and Tables

**Table 1:** Distribution of Specific Binding Ratios (SBR), Asymmetry Indices, and Interregional Ratios Across Baseline- and Q.Clear-DICOM. The yellow-highlighted columns represent subregions from the baseline-DICOM dataset with an average Specific Binding Ratio (SBR) value of 1 or higher. Subregions are arranged in descending order based on their average values in the baseline-DICOM. The lower part of the table summarizes asymmetry indices and interregional ratios. The indices and ratios are listed in random order.

		Baseline-DICOM					Q.Clear-DICOM					
	Total SBR	Min	Max	Average	Stdev	Median	Min	Max	Average	Stdev	Median	*p* Value
	Putamen ventral anterior (VA)	0.94	6.61	**3.62**	1.15	3.72	1.02	7.84	4.25	1.39	4.35	<0.001
	Ventral striatum	0.75	5.21	**3.22**	0.90	3.25	0.92	6.42	3.96	1.11	4.00	<0.001
	Putamen dorsal anterior (DA)	0.61	6.53	**3.19**	1.25	3.34	0.60	7.82	3.72	1.52	3.90	<0.0001
	Putamen	0.60	5.94	**2.94**	1.18	3.11	0.61	7.33	3.51	1.48	3.68	<0.0001
Areas average	Caudate DA	0.25	5.44	**2.90**	1.00	2.93	0.28	7.18	3.70	1.31	3.71	<0.001
SBR > 1	Dorsal striatum	0.47	5.69	**2.89**	1.06	2.96	0.50	7.20	3.56	1.36	3.59	<0.001
(base-DICOM)	Caudate VA	0.17	5.11	**2.88**	0.92	2.84	0.26	6.61	3.69	1.19	3.66	<0.001
	Caudate nucleus	0.16	5.15	**2.79**	0.93	2.81	0.23	6.93	3.64	1.24	3.65	<0.001
	Caudate body	0.03	5.17	**2.69**	0.92	2.70	0.06	7.20	3.62	1.25	3.55	<0.001
	Putamen dorsal posterior (DP)	0.26	5.46	**2.60**	1.21	2.86	0.24	6.85	3.13	1.53	3.45	<0.0001
	Putamen VP	0.33	5.36	**2.49**	1.18	2.70	0.32	6.96	3.09	1.55	3.28	<0.0001
	Caudate tail	0.00	4.27	**2.35**	0.81	2.34	0.04	6.09	3.36	1.19	3.30	<0.001
	Ant Pallidum	0.48	1.53	**0.94**	0.21	0.94	0.40	1.37	0.83	0.18	0.82	<0.001
	Substantia nigra	0.40	1.41	**0.92**	0.20	0.93	0.49	1.97	1.12	0.24	1.13	<0.001
Areas average	Pallidum	0.39	1.36	**0.84**	0.19	0.84	0.29	1.11	0.70	0.15	0.70	<0.001
SBR < 1	Dorsal raphe nucleus	0.01	1.41	**0.78**	0.27	0.83	0.08	1.72	0.85	0.30	0.90	<0.0001
(base-DICOM)	Post Pallidum	0.28	1.23	**0.71**	0.18	0.72	0.17	0.87	0.54	0.12	0.54	<0.0001
	Subthalamic nucleus	0.18	1.01	**0.67**	0.15	0.66	0.28	1.34	0.77	0.17	0.75	<0.001
	Locus coeruleus	0.00	0.74	**0.42**	0.15	0.44	0.07	0.93	0.53	0.18	0.55	<0.0001
Striatal asymmetry index Caudate asymmetry index Putamen asymmetry index		−53.24	45.42	4.00	15.13	5.23	−43.03	49.02	4.77	15.54	6.12	<0.0001
		−93.14	238.86	7.76	22.77	6.87	−66.03	231.80	9.16	21.82	8.72	<0.0001
		−52.86	55.87	2.57	18.59	4.62	−55.16	59.54	2.74	19.72	5.41	<0.0001
Putamen-caudate ratio Caudate-putamen ratio Anterior–posterior putamen ratio		−13.27	2.88	0.98	1.14	1.12	−11.54	2.38	0.89	1.00	1.03	<0.0001
		0.15	2.18	1.03	0.35	0.89	0.19	2.49	1.14	0.42	0.97	<0.0001
		1.09	3.55	1.52	0.47	1.28	1.03	4.11	1.50	0.54	1.23	<0.0001

## Data Availability

The datasets used and/or analyzed during the current study are available from the corresponding author on reasonable request.
